# Latent tuberculosis infection prevalence in rural Madagascar

**DOI:** 10.1093/trstmh/traa054

**Published:** 2020-11-06

**Authors:** Gouri Sadananda, Astrid M. Knoblauch, Andry Andriamiadanarivo, Kimmerling Razafindrina, Ideal Ambinintsoa, Roger Mario Rabetombosoa, Christine E. Pando, Lai Yu Tsang, Peter M. Small, Niaina Rakotosamimanana, Simon Grandjean Lapierre

**Affiliations:** a Case Western Reserve University, 10900 Euclid Ave, Cleveland, OH 44106, USA; b Institut Pasteur de Madagascar, Ambatofotsikely, Antananarivo 101, Madagascar; c Swiss Tropical and Public Health Institute, P.O. Box, 4051 Basel, Switzerland; d University of Basel, P.O. Box, 4003 Basel, Switzerland; e Centre ValBio Research Station, BP 33 Ranomafana, Ifanadiana, Madagascar; f Stony Brook University, Global Health Institute, 100 Nicholls Road, Stony Brook, NY 11794, USA; g Stony Brook University, School of Medicine, 101 Nicholls Road, Stony Brook, NY 11794, USA; h Immunopathology Axis, Centre de Recherche du Centre Hospitalier de l’Université de Montréal, 900 rue Saint-Denis, Montréal H2X 3H8, Canada; i Microbiology, Infectious Diseases and Immunology Department, Université de Montréal, 2900 Boulevard Edouard Montpetit, Montreal H3T 1J4, Canada

**Keywords:** HIV, latent TB infection, Madagascar, TB, tuberculin skin test

## Abstract

**Background:**

Understanding latent *Mycobacterium tuberculosis* infection (LTBI) prevalence is crucial for the design of TB control strategies. There are no data on LTBI in rural Madagascar.

**Methods:**

Tuberculin skin tests were performed in 98 adults aged >15 y in five rural villages in the Ifanadiana district, Madagascar.

**Results:**

Of adults, 78.6% were positive for LTBI, ranging between 28.6% and 95.0% among villages. The majority (65.3%) showed an induration reaction of >15 mm.

**Conclusions:**

LTBI prevalence is high in rural Madagascar. Long-term TB control strategies including LTBI testing and treatment must account for high and heterogeneous prevalence in remote, underdeveloped areas.

## Introduction

An estimated 1.7 billion people are latently infected with *Mycobacterium tuberculosis* (MTB) globally, of whom 5–10% are expected to develop TB in their lives.^1,2^ This represents an immense reservoir. Testing and treatment of latent MTB infection (LTBI) is therefore increasingly considered as part of multi-intervention elimination strategies for TB.^3^ In rural areas of Madagascar, where >60% of the country’s population live, no data on LTBI prevalence are available.

## Materials and methods

In June 2018, a cross-sectional study was conducted to assess the prevalence of LTBI in five rural villages of Ifanadiana district in south-central Madagascar. This area was conveniently selected given other ongoing TB trials, including active case finding-based TB prevalence studies. TB case finding was piloted during the 7 mo preceding this study and active TB cases were found in all five selected villages. From active TB index cases, household and non-household contacts as well as randomly selected community members aged ≥15 y were included. Contacts were defined as per WHO criteria, with household contacts living under the same roof, non-household contacts spending significant time in the same room on a daily basis and community members being unrelated to known TB index cases. Tuberculin skin tests (TSTs) using purified tuberculin (Sanofi Pasteur Inc., Shiftwater, PA, USA) were used to diagnose LTBI. According to Center for Disease Control guidelines, reactions >5 mm were considered positive for household contacts and HIV-infected individuals. Reactions >10 mm were considered positive for all other individuals.

In addition, participants were medically screened and tested using GeneXpert MTB/RIF (rifampicine) assay (Cepheid, Sunnyvale, CA, USA) if presenting active TB compatible symptoms. All participants were counselled and tested for HIV infection using HIV 1/2 combo rapid point of care assay (Alere, Waltham, MA, USA).

## Results

A total of 98 participants were recruited. The mean age was 36.3 y and 44.9% of the participants were men.

Of the 98 participants, 77 (78.6%) had a positive TST (Table 1). All positive participants showed an induration reaction >10 mm whereas the majority showed an induration >15 mm (n=64; 65.3%). Considerable variation in prevalence existed among villages ranging between 28.6% and 95.0%. The majority of participants presented with a Bacille Calmette-Guerin (BCG) scar (n=80; 81.6%) but there was no significant difference in TST positivity based on vaccination status. Among latently infected participants, eight (8.2%) reported experiencing prior active pulmonary TB infection. Positivity rates were not lower in the seven community members (100.0%) than in the 56 non-household contacts (74.7%) or in the 14 household contacts (87.5%).

Upon inclusion in the study, 57 participants (58.2%) presented symptoms compatible with active pulmonary TB such as chronic cough and underwent molecular testing with GeneXpert. No one was found to have a GeneXpert positive sputum. All participants tested negative for HIV.

## Discussion

Our data showed a high prevalence (78.6%) of LTBI in this rural setting of Madagascar. Comparable results were found in the capital Antananarivo, where a 2015 survey among adults (aged >15 y) found rates of LTBI of 76.8% in household contacts and 52.1% in community controls.^4^ Infection rates of community controls were higher in our study (100.0%), potentially reflecting the increased proximity in remote communities compared with urban populations.

Sampling for this study took place in villages where active TB cases had previously (i.e. within 7 mo prior to the study) been diagnosed. Given the crowded living conditions and the applied sampling strategy (i.e. mostly TB contacts), the LTBI prevalence could be overestimated. However, active case finding confirmed the presence of pulmonary TB cases in 15 out of 61 (24.6%) villages visited in this district, suggesting overall high TB incidence. Consequently, a high LTBI rate is to be expected.

Interpretation of TST results can be limited by its interaction with BCG vaccination whereby postinfancy and booster vaccinations can lead to false-positive TST results.^2^ According to national policy, every newborn should receive a single dose at birth and, in our study population, 81.6% of participants harboured a vaccination scar. Although the timing of vaccination could not be assessed in this adult study population, BCG vaccination should not have biased the current findings if performed according to national guidelines.

According to 2018 WHO LTBI guidelines, HIV-negative household contacts (all ages) of confirmed TB patients ‘should’ or ‘may’ receive TB-preventive treatment in high-burden TB countries.^3^ This policy is not yet implemented in rural Madagascar and introducing prolonged therapy for healthy individuals in these remote areas promises to be a challenging undertaking. However, with this immense latency reservoir, active TB cases can emerge anywhere at any time. Prediction scores were previously developed to assess TB reactivation risks among TB household contacts.^5^ In this study, given the high prevalence of LTBI, the high social relatedness of study participants and the relative uniformity of sociodemographic and economic factors, no questionnaire-based score could be developed to predict LTBI infection status.

The current data underscore the importance of questions on how to tackle LTBI, in particular in remote and underdeveloped areas of countries facing challenges in TB control such as Madagascar. Our data are insufficient to inform a systematic LTBI treatment programme, but together with data from collateral active case finding efforts in the same area, they provide evidence to support future transmission and elimination modelling studies. This also emphasises the need for further work on the relationship between age of exposure and the potential value of interventions such as immunisation or household, school entry and maternity TB screening.

## Conclusions

LTBI prevalence is high in rural Madagascar. Long-term TB control strategies including LTBI testing and treatment must account for this high prevalence and the complexities of implementing systematic LTBI-related interventions in remote and isolated areas. Underlying causes and risk factors of TB (e.g. poverty, overcrowding, undernutrition and indoor air pollution) must be addressed concomitantly.

## Figures and Tables

**Table 1.** Tuberculin skin testing results, Ifanadiana district, Madagascar, 2018

		Quantitative TST result (n, %)				TST-positive	
	N	<5 mm	5–9.9 mm	10–14.9 mm	>15 mm	N	% (95% CI)
Village							
Ambalatabaka	23	2 (8.7)	0 (0.0)	4 (17.4)	17 (73.9)	21	91.3 (72.0 to 98.9)
Ambohimahasoa I	8	2 (25.0)	0 (0.0)	1 (12.5)	5 (62.5)	6	75.0 (34.9 to 96.8)
Ambohimahasoa II	20	1 (5.0)	0 (0.0)	3 (15.0)	16 (80.0)	19	95.0 (75.1 to 99.9)
Ampasimpotsy	40	11 (27.5)	0 (0.0)	5 (12.5)	24 (60.0)	29	72.5 (56.1 to 85.4)
Antanivelona	7	5 (71.4)	0 (0.0)	0 (0.0)	2 (28.6)	2	28.6 (3.7 to 71.0)
Exposure group							
Household TB contact	16	2 (12.5)	0 (0.0)	2 (12.5)	12 (75.0)	14	87.5 (61.7 to 98.4)
Non-household TB contact	75	19 (25.3)	0 (0.0)	10 (13.3)	46 (61.3)	56	74.7 (63.3 to 84.0)
Community member	7	0 (0.0)	0 (0.0)	1 (14.3)	6 (85.7)	7	100.0 (59.0 to 100.0)
Presenting a BCG vaccination scar							
Yes	80	4 (22.2)	0 (0.0)	3 (16.7)	11 (61.1)	63	78.8 (68.2 to 87.1)
No	18	17 (21.3)	0 (0.0)	10 (12.5)	53 (66.3)	14	77.8 (52.4 to 93.6)
Total	98	21 (21.4)	0 (0.0)	13 (13.3)	64 (65.3)	77	78.6 (69.1 to 86.2)

BCG, Bacille Calmette-Guérin; TB, tuberculosis; TST, tuberculin skin test.
